# Recombinant outer membrane vesicles carrying *Chlamydia muridarum* HtrA induce antibodies that neutralize chlamydial infection in vitro

**DOI:** 10.3402/jev.v2i0.20181

**Published:** 2013-05-06

**Authors:** Erika Bartolini, Elvira Ianni, Elisabetta Frigimelica, Roberto Petracca, Giuliano Galli, Francesco Berlanda Scorza, Nathalie Norais, Donatello Laera, Fabiola Giusti, Andrea Pierleoni, Manuela Donati, Roberto Cevenini, Oretta Finco, Guido Grandi, Renata Grifantini

**Affiliations:** 1 Novartis Vaccines and Diagnostics, Siena, Italy; 2 Novartis Vaccines and Diagnostics, Cambridge, MA, USA; 3 Department of Evolutionary Biology, University of Siena, Siena, Italy; 4 Externautics S.p.A., Siena, Italy; 5 Microbiology Section, DESOS-University of Bologna St. Orsola Hospital, Bologna, Italy

**Keywords:** neutralizing antibodies, outer membrane vesicles, Chlamydia, DO serine protease, Chlamydia trachomatis

## Abstract

**Background:**

Outer membrane vesicles (OMVs) are spheroid particles released by all Gram-negative bacteria as a result of the budding out of the outer membrane. Since they carry many of the bacterial surface-associated proteins and feature a potent built-in adjuvanticity, OMVs are being utilized as vaccines, some of which commercially available. Recently, methods for manipulating the protein content of OMVs have been proposed, thus making OMVs a promising platform for recombinant, multivalent vaccines development.

**Methods:**

Chlamydia muridarum DO serine protease HtrA, an antigen which stimulates strong humoral and cellular responses in mice and humans, was expressed in Escherichia coli fused to the OmpA leader sequence to deliver it to the OMV compartment. Purified OMVs carrying HtrA (CM rHtrA-OMV) were analyzed for their capacity to induce antibodies capable of neutralizing Chlamydia infection of LLC-MK2 cells *in vitro*.

**Results:**

CM rHtrA-OMV immunization in mice induced antibodies that neutralize Chlamydial invasion as judged by an *in vitro* infectivity assay. This was remarkably different from what observed with an enzymatically functional recombinant HtrA expressed in, and purified from the *E. coli* cytoplasm (CM rHtrA). The difference in functionality between anti-CM rHtrA and anti-CM rHtrA-OMV antibodies was associated to a different pattern of protein epitopes recognition. The epitope recognition profile of anti-CM HtrA-OMV antibodies was similar to that induced in mice during Chlamydial infection.

**Conclusions:**

When expressed in OMVs HtrA appears to assume a conformation similar to the native one and this results in the elicitation of functional immune responses. These data further support the potentiality of OMVs as vaccine platform.

Outer membrane vesicles (OMVs) are closed spheroid particles of a heterogeneous size (10–300 nm in diameter) released from all Gram-negative bacteria studied so far. Several experimental evidences indicate that OMVs are generated through a “budding out” of the bacterial outer membrane (OM) and, consistent with this, the majority of their components are represented by lipopolysaccharide (LPS), glycerophospholipids, OM proteins and periplasmic proteins (1).

It has been proposed that OMV release is an essential step for bacteria to rapidly adapt to variations in the external environment. These variations usually require the expression of a number of surface-associated proteins that have to replace the existing ones, and the release of OMVs appears to be an efficient strategy to achieve OM remodelling (2). However, a multitude of other functions have been attributed to OMVs, including toxins and virulence factors delivery to host cells, interspecies and intraspecies cell-to-cell cross-talk, biofilm formation, genetic transformation and defence against host immune responses (2, 3).

OMVs purified from several pathogens, including *Neisseria, Salmonella, Pseudomonas, Vibrio cholerae Burkholderia* and *E. coli* (4–8), induce potent protective immune responses against the pathogens they are derived from, and anti-*Neisseria* OMV-based vaccines are already in the market and have shown to be very effective (9). Such remarkable protection is attributed not only to the presence of the correct immunogenic antigens but also to the fact that OMVs carry most of the pathogen-associated-molecular patterns (PAMPs) which play a key role in stimulating innate immunity and promoting adaptive immune responses. Because of this “built-in-adjuvanticity” feature, several groups have reported strategies to deliver heterologous proteins to OMVs, with the ultimate objective to develop novel effective vaccines (10–14).

In this work, we provide an additional example of the potentiality of OMVs as vaccine platform.

One of our research interests is the development of a *Chlamydia trachomatis* vaccine. In this context, we have recently applied reverse vaccinology and protein array technologies to identify *Chlamydia* antigens which could induce cell-mediated and humoral immune responses (15). It is generally accepted that *Chlamydia* immunity is mediated by both *Chlamydia*-specific Th1 CD4^+^ T cells and neutralizing antibodies (16). A 4 antigen combination was selected that provides robust protection in the mouse model (15). One of the 4 selected antigens is the DO serine protease HtrA, which is particularly unique in that it induces both high antibody titres and IFNγ^+^/CD4^+^ T cells during natural infection in human beings (Grifantini, personal communication) and during experimental infection in mice. HtrA is a highly conserved protein that belongs to the sophisticated and essential system cells use to reduce the amount of unfolded or aggregated proteins (17). In Gram-negative bacteria, HtrA localizes in the periplasm, and it has been shown to associate with phospholipids and cellular membranes (18, 19). As is the case for its *E. coli* homologue DegP (17, 19), Chlamydial HtrA assembles in an inactive trimer which upon temperature induction forms high-molecular-weight 12- and 24-mer multimeric complexes with proteolytic and chaperon-like activities (20, 21). Furthermore, recent studies show that HtrA is released into the cytosol of infected cells and suggest that the protein plays a key role in *Chlamydia* pathology through a mechanism that involves the proteolytic alteration of host cell signalling pathways (22).

Here, we show that both *C. muridarum* (CM) HtrA and *C. trachomatis* (CT) HtrA are exposed on the surface of elementary bodies (EBs). Furthermore, we show that CM HtrA expressed in *E. coli* with a leader sequence for secretion is incorporated into OMVs. When CM rHtrA-bearing OMVs (CM rHtrA-OMV) are used to immunize mice, anti-HtrA-specific antibodies are induced that neutralize *Chlamydia* infection as judged by an in vitro infectivity assay. The neutralizing activity is not observed with antibodies elicited by an enzymatically functional recombinant HtrA (CM rHtrA) expressed in, and purified from, the *E. coli* cytoplasm. We also provide evidence that the difference in functionality between anti-CM rHtrA and anti-CM rHtrA-OMV antibodies is associated with a different profile of protein epitope recognition.

To the best of the authors’ knowledge, this is the first demonstration that, by virtue of their ability to carry properly folded membrane-associated proteins, recombinant OMVs can be exploited to induce functional immune responses that otherwise would be difficult to elicit. Altogether, these data reinforce the notion that OMVs represent a promising platform for the development of new effective vaccines.

## Materials and methods

### Bacterial strains, cultures and reagents

*C. muridarum* strain Nigg and CT serovar D strain D/UW-3/CX were grown on confluent monolayers of LLC-MK2 (ATCC CCL7) in Earle's minimal essential medium (EMEM) as described previously (23, 24). Purification of CT and CM EBs was carried out by renografin density gradient centrifugation as described previously (23). *Escherichia coli* BL21(DE3) was grown aerobically in Luria broth (LB) medium (Difco) at 37°C. When appropriate, ampicillin (Amp, 100 µg/mL), isopropyl-β-d-galactopyranoside (IPTG; 1 mM) and kanamycin (Kana, 30 µg/mL) were added to the medium. Unless specified, all chemicals used in this study were purchased from Sigma. Restriction enzymes and DNA modification enzymes were from New England Biolabs. Fluorescence-activated cell sorter (FACS) reagents and antibodies were from BD Biosciences.

### Gene cloning and protein purification

To produce recombinant proteins CT and CM HtrA (CT823 and TC0210), CT MOMP (CT681) and CM MOMP (TC0052), genes were polymerase chain reaction (PCR) amplified from CT and CM chromosomal DNA using specific primers annealing at the 5’ and 3’ ends of either gene and cloned into plasmid pET21b+ (Invitrogen), so as to fuse a 6-histidine tag sequence at the 3’ end. Cloning and purification of His fusions were performed as already described (15, 24). CT MOMP and CM MOMP expressed as His fusion proteins were purified from the insoluble protein fraction, while CT and CM rHtrA expressed as His fusion proteins were purified from the soluble protein fraction according to the manufacturer's procedure.

### Construction of BL21(DE3) ΔtolR deletion mutant

The BL21(DE3) Δ*tolR* mutant was produced by replacing *tolR* coding sequence with a kanamycin-resistance cassette as described (25). Essentially, the *tolR* upstream and downstream regions were fused to the kanamycin-resistance gene (*kmr*) using a 3-step PCR protocol (25). The linear fragment obtained, in which the *kmr* gene was flanked by the tolR upstream and downstream regions, was used to transform the recombination-prone BL21(DE3) *E. coli* strain, and Δ*tolR* mutants were selected by plating transformed bacteria on Luria–Bertani (LB) plates containing 30 µg/mL of kanamycin. Recombination-prone BL21 (DE3) cells were produced using the highly proficient homologous recombination system (red operon) (25, 26). The deletion of the *tolR* gene was confirmed by PCR amplification of genomic DNA using pairs of primers specifically annealing to *tolR* and to *kmr* gene, as described (25).

### Expression of CM rHtrA in BL21(DE3) ΔtolR mutant strain

In order to address CM rHtrA (TC0210) on the OM of *E. coli* mutant strains, the CM *htrA* gene was amplified with specific pairs of primers so as to replace the native leader peptide with the *E. coli* OmpA leader peptide (MKKTAIAIAVALAGFATVAQA) and to add a 6-histidine tag sequence at the 3’ terminus. The PCR product was inserted in pET21b+ (Invitrogen) plasmid using the polymerase incomplete primer extension (PIPE) cloning method (27) and the resulting plasmid was used to transform *E. coli* HK100 cells, made CaCl_2_ competent after several successive washes in cold MgCl_2_ and CaCl_2_ solutions (27).

Cells were plated on LB containing 100 µg/mL of ampicillin (LB-Amp) at 37°C overnight and positive clones were selected by PCR analysis. Plasmid DNA was isolated and used to transform BL21(DE3) Δ*tolR* strain, made electro-competent by 3 washing steps in cold water. The resulting BL21(DE3) Δ*tolR*(pET-*htrA*) strain was grown in LB-Amp to an OD_600_ of 0.6 and induced with 1 mM IPTG for 2 h. *C. muridarum* rHtrA expression was verified in total protein extracts and culture supernatant of the BL21(DE3) Δ*tolR*(pET-*htrA*) strain by SDS-PAGE and Western blot, using the BL21(DE3) Δ*tolR*(pET) strain containing the empty pET21b+ vector as the negative control.

### OMV preparation and characterization

#### OMV preparation

Culture media of the BL21(DE3) Δ*tolR*(pET-*htrA*) and BL21(DE3) Δ*tolR*(pET) strains were filtered through a 0.22-µm pore size filter (Millipore, Bedford, MA, USA). The filtrates were clarified by centrifugation and subjected to high-speed centrifugation (200,000*g* for 2 h), and the pellets containing the OMVs were washed with phosphate buffer saline (PBS) and finally resuspended with PBS (25).

#### Laser-scattering analysis

Size distribution profile of OMVs was determined by dynamic light scattering based on laser diffraction method using Malvern Zetasizer (Version 6.0, Malvern, UK). The OMV diameter was determined after dilution in PBS (1/10) and by measuring the back scattering intensity (175°) at 25°C. Three measurements (15 experimental runs for each measurement) were averaged to determine the vesicle size.

#### Immunogold electron microscopy

Immunogold electron microscopy of OMV preparation was performed as previously described (25). Briefly, purified OMVs were charged onto Formvar-coated nickel grids, incubated for 10 min and incubated for 30 min in PBS containing 1% bovine serum albumin (BSA) (blocking buffer). Samples were then labelled for 1 h with polyclonal anti-CM rHtrA rabbit IgG, previously pre-adsorbed on empty OMVs and diluted in blocking buffer at a concentration of 10 µg/mL. After washing with blocking buffer, samples were subsequently incubated with a 1:40 dilution of a secondary goat anti-rabbit immunoglobulin G conjugated to 5-nm gold particles (BB International). Finally, samples were washed and stained with 1% (wt/vol) uranyl acetate in water before analysis in a TEM FEI Tecnai G2 spirit operating at 100 kV and equipped with a CCD camera (Olympus SIS Morada).

### OMV protein digestion and mass spectrometry analysis

OMV protein digestion was carried out using trypsin in 50 mM ammonium bicarbonate containing 0.1% (wt/vol) RapiGest™ SF Protein Solubilization Reagent (Waters) and 5 mM dithiothreitol (DTT). The resulting peptides were identified by LC/MS-MS. Peptides were separated by nano-LC on a NanoAcquity UPLC system (Waters, Milford, MA, USA) connected to a Q-ToF Premier ESI mass spectrometer equipped with a nanospray source (Waters). Samples were loaded onto a NanoAcquity 1.7-µm BEH130 C18 column (75 µm×25 mm; Waters) through a NanoAcquity 5-µm Symmetry^®^ C18 trap column (180 µm×20 mm; Waters). Peptides were eluted with a 120-min gradient of 2–40% acetonitrile, 0.1% formic acid solution at a flow rate of 250 nL/min. The eluted peptides were subjected to an automated data-dependent acquisition using the MassLynx software, version 4.1 (Waters) where an MS survey scan was used to automatically select multicharged peptides over the *m/z* ratio range 500–2,000 for further tandem mass spectrometry (MS/MS) fragmentation. Up to 8 different peptides were individually subjected to MS/MS fragmentation following each MS survey scan. After data acquisition, individual MS/MS spectra were combined, smoothed and centroided using ProteinLynx, version 3.5 (Waters) to obtain the peak list file. The Mascot Daemon application (Matrix Science Ltd., London, UK) was used for the automatic submission of data files to in-house licensed Mascot, version 2.2.1, running on a local server. Protein identification was carried out from the generated peak list using the Mascot program (Mascot server version 2.2.01, Matrix Science), using a public database (National Center for Biotechnology Information non-redundant (NCBInr), Gram-negative, release June 19, 2007; 5,043,617 sequences) or a from database containing protein sequences deduced from the available sequenced *E. coli* BL21 genome. The Mascot search parameters were set to (a) 2 as the number of allowed missed cleavages (only for trypsin digestion); (b) methionine oxidation and glutamine and asparagine deamidation as variable modifications; (c) 0.3 Da as the peptide tolerance; and (d) 0.3 Da as the MS/MS tolerance. Only significant hits were considered as defined by the Mascot scoring and probability system.

Protein localization was predicted using the software LipoP (version 1.0; Center for Biological Sequence Analysis, Technical University of Denmark (http://www.cbs.dtu.dk/services/LipoP)) and Psortb (version 3.0.2; Simon Fraser University, British Columbia (http://www.psort.org/psortb)).

### Preparation of anti-CM rHtrA and anti-CM rHtrA-OMV antibodies

Mouse antisera were generated and treated as described (24). Briefly, groups of 6-week-old female BALB/c mice purchased from Charles River Laboratories (10–15 mice/group) were immunized intramuscularly (im) at 2-week intervals with 3 doses (20 or 1 µg) of purified CM rHtrA or 50 µg of CM rHtrA-OMV formulated with alum hydroxide as an adjuvant at the final concentration of 3 mg/mL. Groups of mice that received the adjuvant alone or “empty” OMVs not expressing HtrA were included as the negative control. Immune sera were prepared from blood samples collected on day 60 and pooled before use.

Rabbit anti-CM rHtrA IgGs were obtained as follows: 2 female New Zealand rabbits (approximately 2 kg) were immunized subcutaneously at days 1, 20 and 35 with 3 doses (100 µg) of CM rHtrA formulated with equal volumes of Freund's adjuvant. Two weeks after the last immunization rabbits were bled, sera pooled and the total IgGs were purified by affinity chromatography on Protein A Sepharose Fast Flow (GE Healthcare) following the manufacturer's instructions. All procedures were approved by the National Health Institution and Novartis Vaccines Animal Care and Ethical Committee. Unless otherwise specified, before immunological analysis immune sera were incubated overnight at 4°C with nitrocellulose strips adsorbed with an *E. coli* BL21 total protein extract (*E. coli* contaminants), in order to reduce the amount of antibodies elicited by contaminating *E. coli* antigens.

### Enzyme-linked immunosorbent assay

Serum IgG titres directed to CM rHtrA were assayed by enzyme-linked immunosorbent assay (ELISA). Individual wells of micro-ELISA plates (Nunc Maxisorp) were coated with 1 µg of CM rHtrA in PBS (pH 7.4) at 4°C overnight. The plates were washed, treated for 1 h at 37°C with PBS–1% BSA, and 100 µL aliquots of antisera at different serial dilutions in PBS–0.1% Tween 20 were added to the wells. After incubation for 2 h at 37°C, plates were again washed and incubated for 1 h at 37°C with alkaline phosphatase-conjugated goat anti-mouse IgG (Sigma) diluted at 1:2,500 in PBS–Tween 20. Later, 100 µL of PNPP substrate (Sigma) was added to the samples and incubated for 30 min at room temperature and optical densities (ODs) were read at 405 nm. Serum antibody titres were defined as the reciprocal of the serum dilution yielding an OD value of 0.5.

### Western blot assay

For Western blot analysis, total proteins from purified EBs of CM strain Nigg or CT serovar D strain D/UW-3/CX (2 µg/lane), or OMV preparations or purified rHtrA (20 µg and 200 ng, respectively, unless otherwise stated) were respectively separated by SDS-PAGE (4–12% gel) under reducing conditions and electroblotted onto nitrocellulose membranes. After saturation with PBS–0.1% Tween 20 containing 5% skim milk, the membranes were incubated with the indicated sera diluted at 1:200 in PBS–0.1% Tween 20–3% skim milk (antibody buffer) for 2 h at 37°C. After washing with the antibody buffer, membranes were incubated with a peroxidase-conjugated anti-mouse antibody (diluted at 1:3,000 in the antibody buffer) for 1 h at 37°C. After washing with PBS–0.1% Tween 20, blots were developed using an Opti-4CN Substrate Kit (Bio-Rad). The densitometric analysis of CM rHtrA bands was done with the ImageJ software (National Institutes of Health).

### Flow cytometry assay

Flow cytometry analysis on CT and CM was performed as described previously (24). Purified CM or CT EBs were rendered not infectious by UV treatments. Inactivated EBs (2×10^5^ cells) resuspended in PBS–0.1% BSA were incubated for 30 min at 4°C with anti-CM rHtrA and anti-CM rHtrA-OMV antibodies, using anti-rMOMP antibodies as positive controls (standard dilution: 1:200). As the negative control, anti-CM rHtrA and anti-CM rHtrA-OMV antibodies were also tested on *E.coli* expressing CM rHtrA in the intracellular compartment. After centrifugation and washing with PBS–0.1% BSA, the samples were incubated for 30 min at 4°C with goat anti-mouse IgG, F(ab)'2-specific, conjugated with R-Phycoerythrin (Jackson Immunoresearch Laboratories Inc.). The samples were washed and resuspended in PBS–0.1% BSA and analyzed by a flow cytometer.

For flow cytometry analysis of OMVs, CM rHtrA-OMV and “empty” OMV preparations were incubated for 30 min at 4°C with affinity-purified rabbit anti-CM rHtrA IgG (dilution: 1/100) or with irrelevant purified rabbit IgG and subsequently incubated with an Alexa Fluor 647^®^ fluorescently labelled goat anti-rabbit antibody (dilution: 1/800), without washing steps. Additional controls included OMV staining with the secondary antibody alone. Flow cytometry data were acquired on a FACS Canto^™^ II (BD Biosciences). The settings for acquisition of the samples used detector amplifications not exceeding 370 V to avoid the light laser's noise and adjusting the threshold in order to decrease the noise background. Samples were acquired using the Cell Quest Software (Becton Dickinson, Mountain View, CA, USA) and analyzed using FloJo software v9.1 (Tree Star Inc.). Data significance was elaborated by calculating the Kolmogorov–Smirnov statistics (K–S score) (28).

### In vitro serum neutralization assays

CT and CM neutralisation assays were performed in LLC-MK2 (rhesus monkey kidney) epithelial cell cultures as previously described (24). Briefly, 15 µL of CT EBs or CM EB suspension (3×10^5^ IFU/mL diluted in sucrose phosphate buffer (SP)) were added to serial dilutions of pre-immune and immune sera from vaccinated mice and incubated for 30 min at 37°C on a slowly rocking platform, also including EB samples without serum as the infection control (EBs–SP control). One hundred microlitres of each dilution was then inoculated in duplicate into LLC-MK2 confluent monolayers in a 96-well plate and centrifuged at 750 *g* for 1 h at 37°C. After centrifugation, EMEM containing 10% foetal bovine serum and 1 µg/mL cycloheximide were added. Infected cultures were incubated at 37°C for 48 or 24 h for CT and CM, respectively. After methanol fixation, monolayers were stained with a mouse anti-*Chlamydia* fluorescein-conjugated monoclonal antibody (Merifluor Chlamydia, Meridian Diagnostics, Inc.) and counted for IFUs.

The reduction of infectivity due to EB interaction with the immune sera was calculated as the percentage reduction in mean IFU number as compared to the EBs–SP control. In this calculation, the IFU counts obtained with immune sera were further corrected for background inhibition of infection of the corresponding pre-immune mouse serum. Experimental variability was evaluated by calculating the standard error of measurement (SEM), from 3 titration experiments for each recombinant antigen.

### Peptide spot synthesis

Spot synthesis of 15-mer peptides, overlapping by 10, and covering the mature form of the CM HtrA sequence was performed on 3 amino-polyethyleneglycol-cellulose membranes by an automated spot synthesizer (MultiSynTech, Bochum, Germany) using 9-fluorenylmethoxycarbonyl chemistry and *O*-(benzotriazol-1-yl)-*N*,*N*,*N*,*N*-tetramethyluronium hexafluorophosphate-1,3-diisopropylethylamine activation. After the final synthesis cycle, the side-chain protective groups were removed using a mixture of trifluoroacetic acid–triisobutylsilane–water–dichloromethane.

### Peptide binding assay

Cellulose-bound peptides were soaked in ethanol to prevent hydrophobic interaction between the peptides. Non-specific binding was blocked by incubating cellulose membranes overnight at 4°C with 10 mL of 5% skim milk in PBS containing 0.05% Tween 20. The membranes were incubated with anti-CM rHtrA and anti-CM rHtrA-OMV antibodies and with sera from mice that received 2 sequential infections with CM EBs, for 2 h at 37°C (1/200 dilutions). After washing, membranes were incubated with an HRP-conjugated goat anti-mouse immunoglobulin G (1/1,000 dilution) and developed with Opti 4CN-substrate kit (BIORAD). The analysis was repeated twice with identical results. The staining of the empty OMV antiserum and the secondary antibody on the same panel of peptides was negligible.

### Molecular modelling

The crystal structures of *E. coli* DegP (PDB IDs: 1ky9, 2zle and 3cs0) were used to generate homology models of CM rHtrA. Models were generated using Modeller9v8. Graphical representation shown in Fig. 5C were generated with UCSF Chimera 1.5.1.

## Results

### Antibodies against recombinant HtrA recognize HtrA on the EB surface but do not neutralize Chlamydia infectivity in vitro

One immunological mechanism that can contribute to *Chlamydia* immunity is the elicitation of antibodies which neutralize EB cell entry. A prerequisite for an antigen to induce such antibody-mediated neutralizing activity is its exposure on the EB surface. We previously demonstrated that HtrA is present on the surface of *Chlamydia pneumoniae* EBs (24). Therefore, to establish whether EB surface expression is a general feature in all *Chlamydia* species, thus making HtrA a possible target for neutralizing antibodies, we scanned the surface of both CT and CM EBs using flow cytometry and MS analyses.

**Fig. 1:**
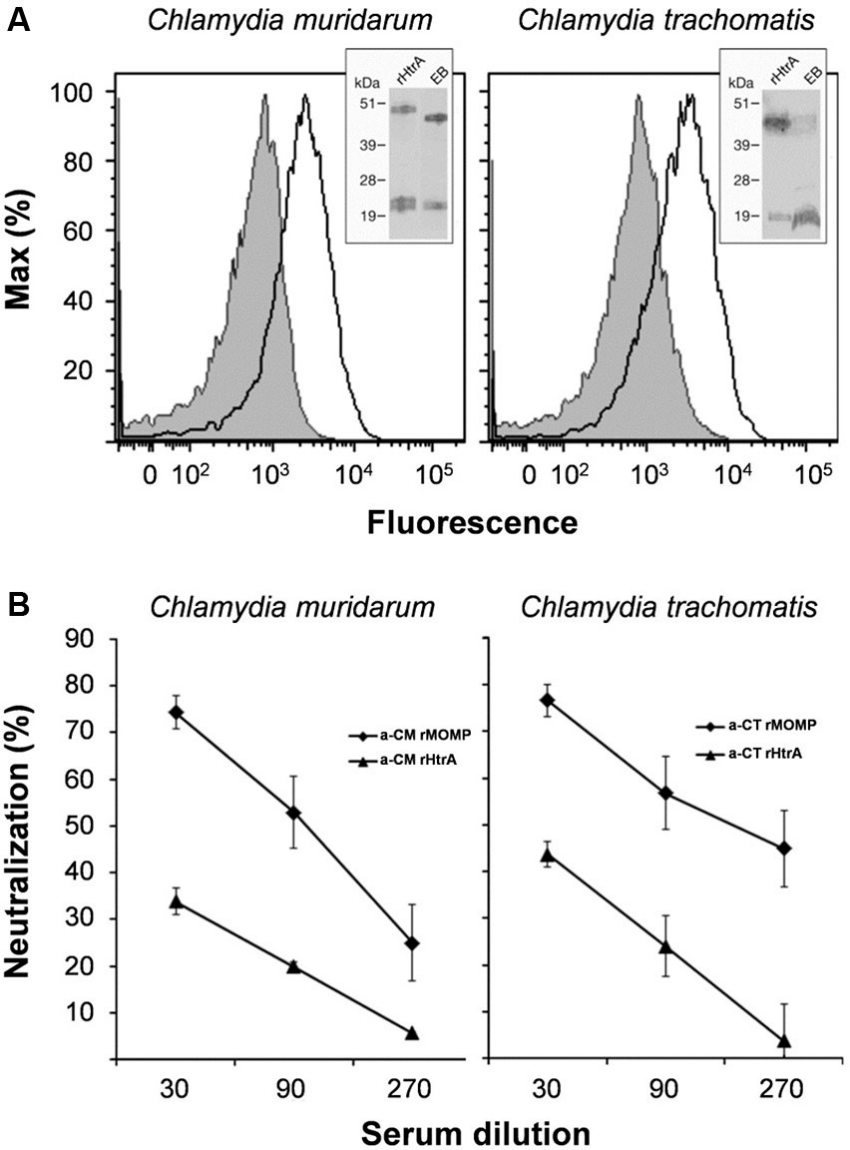
Surface-exposed *Chlamydia* HtrA does not elicit neutralizing antibodies. (A) HtrA *Chlamydia* HtrA is expressed on the EB surface. Purified CM (left panel) and CT (right panel) EBs were incubated with sera raised against CM rHtrA or CT rHtrA (empty peaks) or anti-*E. coli* contaminants (shaded peaks) and the antibody binding on EB surface was detected by flow cytometry using a R-Phycoerythrin-conjugated secondary antibody. Inset panels: Western blot analysis of HtrA expressed by EBs. Total extracts from CM or CT EBs (approximately 10^7^) and purified CM or CT rHtrA were separated on SDS-PAGE, transferred to nitrocellulose membranes and incubated with the corresponding rHtrA antiserum. Molecular weight standards are reported on the left side of each panel. (B) Antibodies elicited by CM rHtrA do not neutralize *Chlamydia* infectivity in vitro. Anti-CM rHtrA and anti-CT rHtrA antibodies were pre-incubated at different dilutions with infectious CM EBs (left panel) and CT EBs (right panel), respectively, and used to infect LLC-MK2 cell monolayers, as compared with EBs treated with corresponding dilutions of pre-immune sera. As positive controls, anti-CM rMOMP and anti-CT rMOMP were also used simultaneously. The antiserum neutralization activity was determined by measuring the reduction of the number of inclusions generated by antibody-opsonized EBs after correction for background inhibition observed with pre-immune sera. The graphs represent the percent neutralization averaged from triplicate experiments, with standard deviations.

Polyclonal antibodies raised against recombinant CM HtrA (CM rHtrA) and CT HtrA (CT rHtrA) purified from the *E. coli* cytoplasm were tested for their ability to bind EBs by flow cytometry. As shown in Fig. 1A, both anti-CM rHtrA and anti-CT rHtrA antibodies stained the surface of whole EB populations, giving a shift in the mean fluorescence peak of approximately 0.7 log with respect to the negative control serum. The same antibodies did not bind the surface of *E.coli* expressing CM rHtrA in the cytoplasm, further confirming the specificity of the EB surface immune-staining (Fig. 1S). HtrA surface localization was also verified by subjecting CT EBs to mild trypsin digestion, so as to remove the protein domains protruding out of the EB surface while preserving EB integrity (29). After digestion, “shaved” EBs were removed by filtration and the proteolytic peptides present in the supernatant were analyzed by MS/MS. The peptide mixture included one specific peptide matching the sequence GENVLLMVSQGDVVR and corresponding to the HtrA region from amino acid 475–489 located at the carboxy-terminus of the protein.

We next assessed the ability of anti-rHtrA antibodies to prevent EB infection of LLC-MK2 cells in vitro. CT and CM EBs were pre-incubated with anti-CT rHtrA and anti-CM rHtrA antibodies, respectively, and then used to infect LLC-MK2 cell monolayers. Inclusions were counted under the microscope after 48- or 24-h-incubation for CT and CM, respectively. Sera against recombinant MOMP of CT and CM (anti-CT rMOMP and anti-CM rMOMP) were used as positive controls of the in vitro neutralisation assay (30, 31). While antibodies against rMOMP were able to neutralize EB infectivity in vitro, anti-rHtrA antibodies only weakly inhibited inclusion formation in LLC-MK2 cells (Fig. 1B) and the IFU reduction did not reach 50%, the threshold value usually used to score a serum as neutralizing. Neutralization ability of anti-rHtrA antibodies was not improved by formulating the protein with different adjuvants, including Freund, alum or LTK63-CpG (data not shown).

**Fig. 2:**
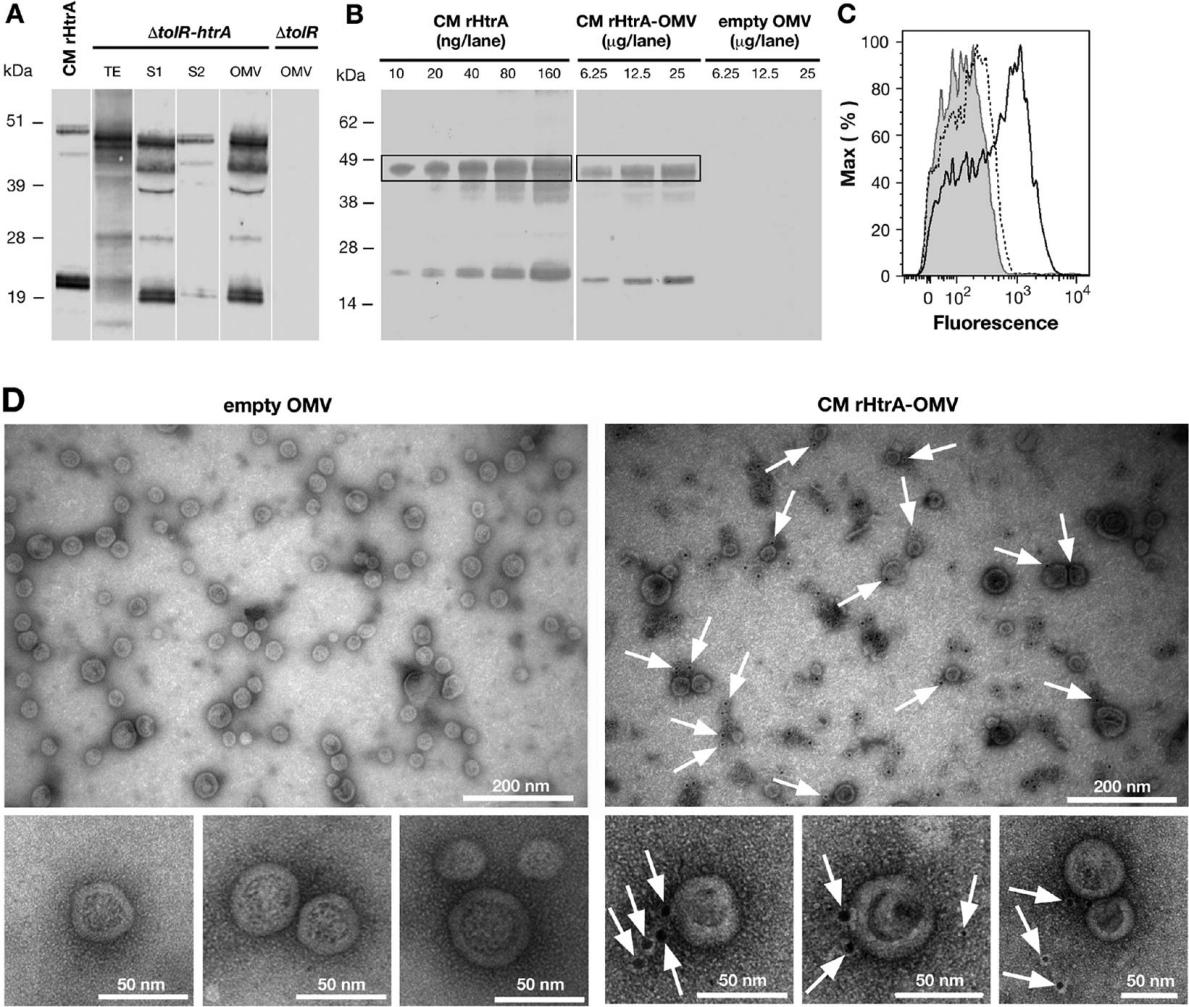
CM rHtrA expressed by *E. coli* BL21(DE3) Δ*tolR*(pET-*htrA*) is associated with the OMV fraction and is partially detected on the OMV surface. (A) Western blot analysis of CM rHtrA expression and compartmentalisation. Mid log cultures of *E. coli* BL21(DE3) Δ*tolR*(pET-*htrA*) (marked as Δ*tolR*(pET-*htrA*)) were induced with 1 mM IPTG for 3 h. CM rHtrA expression was assessed by Western blot in total bacterial extracts (TE), culture supernatant before (S1) and after (S2) OMV purification and in the purified OMV fraction. Each loaded sample corresponded to 5 mL of the original bacterial culture. OMVs purified from the recipient strain *E. coli* BL21(DE3) Δ*tolR*(pET) (empty OMV) and IMAC-purified CM rHtrA were run simultaneously as controls. As shown in the figure, CM rHtrA exists in 2 major protein species of approximately 48 and 20 kDa, representing the full length and a processed form of the protein, as verified by MALDI-TOF analysis. Both bands are also visible in the Δ*tolR*(pET-*htrA*)-derived samples. CM rHtrA bands show a slightly higher molecular weight due to the presence of the histidine tag. Molecular weight markers are on the left. (B) Estimation of CM rHtrA amount present in CM rHtrA-OMVs by comparative Western blot. Different quantities of CM rHtrA-OMVs and CM rHtrA were analyzed by immunoblot and the amount of rHtrA present on CM rHtrA-OMVs was estimated by densitometric analysis of the 48-kDa HtrA band (boxed). (C) FACS analysis of rHtrA surface exposure on CM rHtrA-OMV. Purified CM rHtrA-OMV (empty peak) and “empty” OMVs (shaded peak) were incubated with anti-CM rHtrA antibodies. CM rHtrA-OMV were also incubated with antibodies raised against *E. coli* contaminants (dotted peak) as an additional negative control. The binding was revealed using an Alexa Fluor 647 labelled secondary antibody. (D) Immuno electron microscopy analysis of CM rHtrA-OMV. CM rHtrA-OMVs and empty OMVs were incubated with anti-CM rHtrA antibodies and the presence of rHtrA was detected with a secondary gold-conjugated antibody. Arrows indicate HtrA immunogold staining. Bars represent the image size scale.

We tested whether the inability to induce neutralizing antibodies was due to structural modification of the protein which, when expressed in the *E. coli* cytoplasm, did not assemble in proteolytically active multimers. For this purpose, purified CM rHtrA was subjected to an in vitro protease assay, using BSA as the substrate. The results of this analysis indicated that the protein could almost completely hydrolyse the substrate after 16-h incubation at 37°C (Fig. 2S).

Overall, these results indicate that HtrA expressed in the *E. coli* cytoplasm forms high-molecular-weight complexes and is proteolytically active but is unable to elicit neutralizing antibodies in mice.

### CM rHtrA is found associated with OMVs when expressed fused to the OmpA signal sequence

We next investigated whether by delivering CM HtrA to the periplasmic space of *E. coli*, the protein could be incorporated into OMVs. We were also interested to know whether the protein, similarly to what happens in chlamydial EBs, could reach the *E. coli* OM and therefore be detected on the surface of OMVs.

**Fig. 3:**
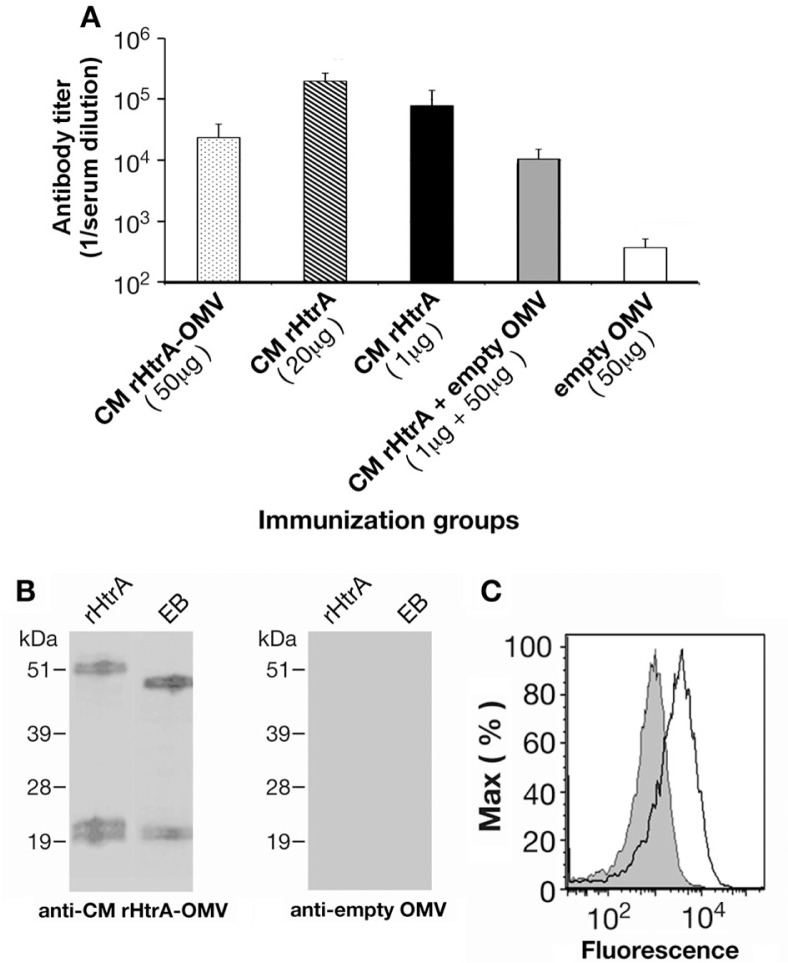
Antibodies raised against rHtrA-OMV recognize *C. muridarum* HtrA and bind the EB surface. (A) HtrA-specific ELISA IgG titres. Sera from mice immunized with different OMV formulations (CM rHtrA-OMV, empty OMV and empty OMV mixed with rHtrA) and with purified rHtrA (doses of 20 and 1 µg) were collected 2 weeks after the third immunization and IgG titres were analyzed by ELISA. Bars represent the mean titres of each mouse group expressed as the reciprocal of the serum dilution yielding an OD value of 0.5. (B) Western blot analysis of CM EBs. Total extracts of CM EBs and purified CM rHtrA were loaded on the gel, transferred to nitrocellulose and immunoblots were probed using antibodies raised against CM rHtrA-OMV (left panel) and empty OMV (right panel). Molecular weight standards are reported on the left of each panel. (C) Flow cytometry analysis of CM EBs. CM EBs were incubated with anti-CM rHtrA-OMV (open peak) and anti-empty OMV antibodies (shaded peak) and surface-bound antibodies were detected by flow cytometer using a R-phycoerythrin-conjugated secondary antibody.

**Fig. 4:**
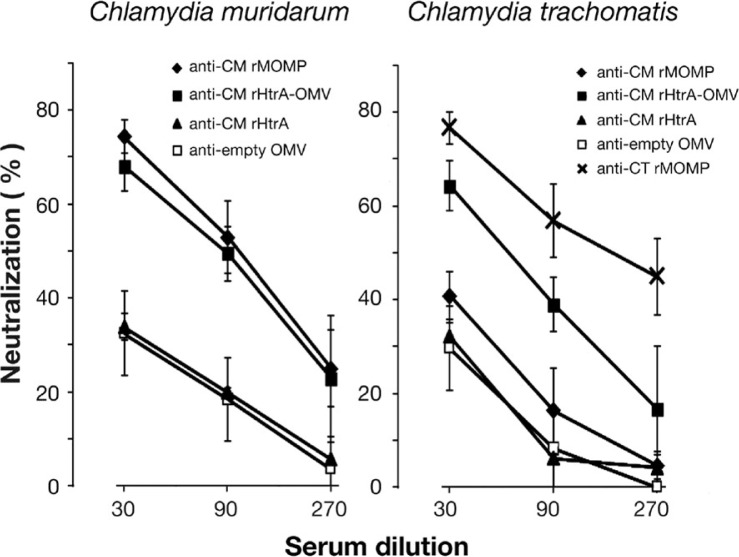
Antibodies directed against CM rHtrA-OMV neutralize *Chlamydia* infectivity in vitro. CM EBs (left panel) and CT EBs (right panel) were opsonized with sera directed against CM rHtrA, CM rHtrA-OMV, “empty” OMV at different dilutions and used to infect LLC-MK2 cell monolayers, in comparison with EBs treated with corresponding dilutions of pre-immune sera. As controls, EBs were also incubated with anti-CM rMOMP and anti-CT rMOMP antibodies. The antiserum neutralization activity was determined by measuring the reduction of the number of inclusions generated by antibody-opsonized EBs after correction for background inhibition observed with pre-immune sera. The graphs present the percent neutralization averaged from triplicate experiments, with standard deviations.

Hence, using the IPTG-inducible expression plasmid pET21, CM rHtrA was expressed fused to the OmpA leader sequence in BL21(DE3) Δ*tolR*, an *E. coli* mutant strain shown to release large quantities of OMVs into the culture supernatant. BL21(DE3) Δ*tolR(*pET-*htrA)* was grown at 0.6OD_600_ and then HtrA expression was induced with 1 mM IPTG (Fig. 3S). After 2-h induction, OMVs were purified from the bacterial culture supernatant by ultracentrifugation and their quality was assessed by light scattering and electron microscopy (EM) to establish the size and homogeneity of OMVs, and by proteome analysis to confirm that most of the OMV proteins belonged to the OM and periplasmic compartment. Malvern Zetasizer and EM analyses indicated that the majority of the vesicles was in the 25–50 nm range, similar to the size of OMVs purified from BL21(DE3) Δ*tolR(*pET) as the control (Fig. 4S).

In the case of proteomic analysis, OMVs purified from BL21(DE3) Δ*tolR*(pET-*htrA*) strain were digested with trypsin and the proteolytic peptides were then analyzed by LC/MS-MS. A total of 29 proteins were identified in the CM rHtrA-OMV preparation, 15 of which were also identified in OMVs of the recipient strain BL21(DE3)Δ*tolR*(pET) (referred as “empty” OMVs). According to the software LipoP (http://www.cbs.dtu.dk/service/LipoP) and Psortb (http://www.psort.org/psortb), of the 29 identified proteins, 26 (89.7%) could be classified either as lipoproteins, OM or periplasmic proteins, while the remaining 3 proteins are predicted as cytoplasmic or have unknown localization (Table IS).

Next, we estimated the presence of CM rHtrA in OMVs. The first demonstration that delivery of CM rHtrA to the periplasm of *E. coli* results in the incorporation of rHtrA in OMVs derived from MS analysis described above. As shown in Table IS, CM rHtrA was detected in the CM rHtrA-OMV preparation, while not in the OMVs from the recipient strain.

Furthermore, CM rHtrA compartmentalization was investigated by Western blot analysis. After cell separation, 500 mL of culture supernatant were ultracentrifuged, the OMV pellet was resuspended in 500 µL of PBS and 5 µL were loaded on SDS-PAGE. Five millilitres of the culture supernatant and of the ultracentrifugation supernatant were TCA-precipitated and loaded simultaneously. As shown in Fig. 2A, CM rHtrA was found in the culture supernatant and the majority of it accumulated in the OMV fraction.

Finally, CM rHtrA quantification was carried out by densitometric analysis of immunoblot. Particularly, different doses of CM rHtrA-OMVs were subjected to Western blot and the intensity of the HtrA band of approximately 48 kDa (Fig. 2B) was compared with the corresponding band of defined amounts of CM rHtrA. This analysis indicated that in 12.5 µg of CM rHtrA-OMVs, approximately 20 ng of CM rHtrA were present corresponding to 0.16% of the total OMV proteins.

**Fig. 5:**
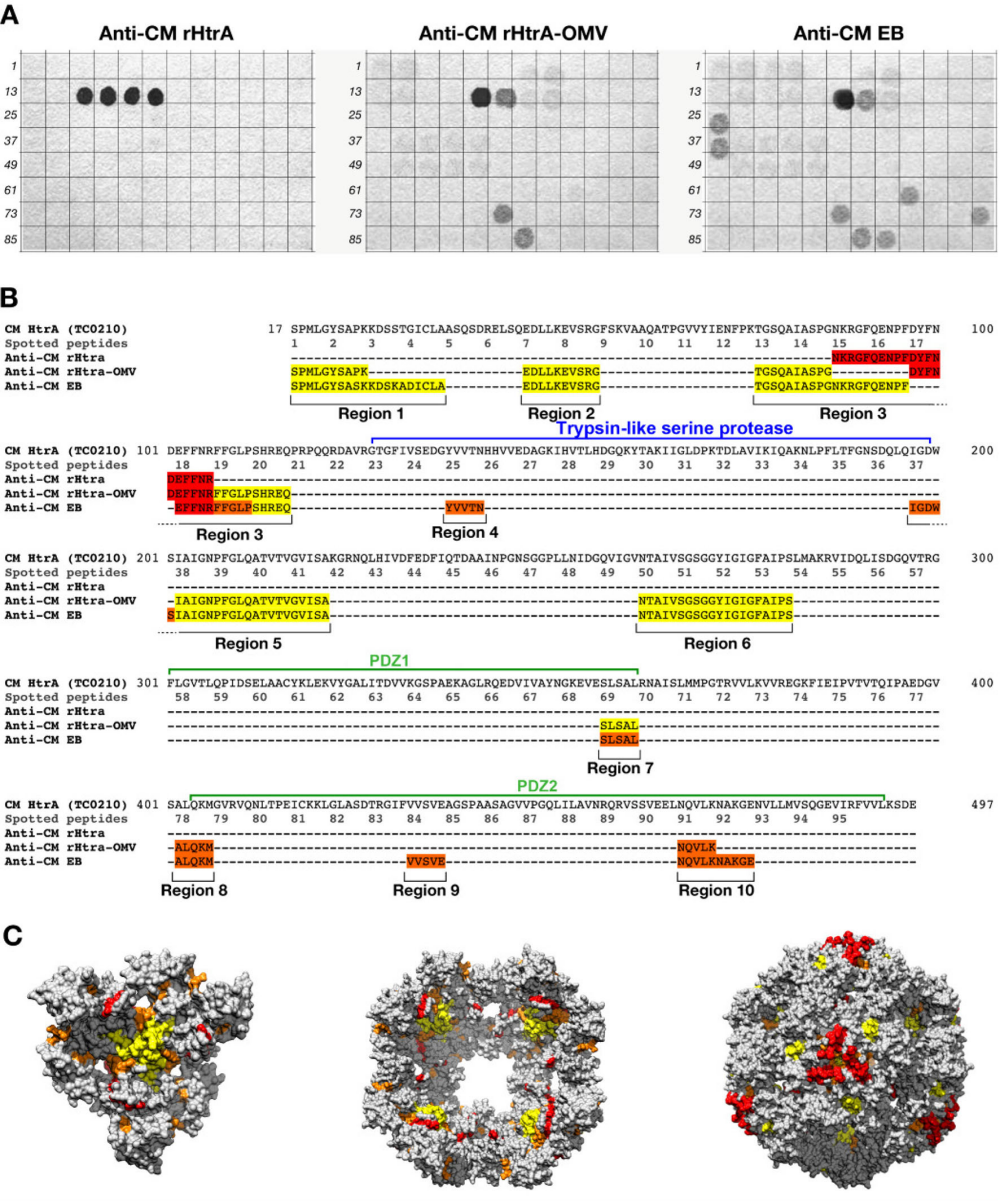
Differential epitope recognition pattern of CM rHtrA and CM rHtrA-OMV antisera. (A) Peptide arrays (96 peptides) were prepared on a cellulose membrane by in situ synthesis of overlapping sequences of 15-residue peptides covering the amino acid sequence of the mature CM HtrA form and tested for binding by immunoblotting with polyclonal antibodies directed against CM rHtrA-OMV (left), CM rHtrA-OMV (centre) and CM EBs (right). Membranes incubated with serum raised against empty OMVs and run simultaneously gave negative staining. Numbers on the left of each blot mark the first peptide of each row of the arrays. (B) Colour code representation of the amino acid regions recognized by the different antisera on the mature from of the CM HtrA protein sequence. The sequences recognized by each antiserum are highlighted according to the staining intensity of spotted peptides: red, high intensity; orange, moderate intensity; yellow, low intensity. (C) Molecular modelling of CM HtrA multimeric forms. The 3-mer basic structure that assembles in multiple copies to form both the bowl-shaped, lipid-associated structures and the spherical soluble structures is shown on the left. The 4-fold symmetry bowl-like structure proposed by Shen and colleagues (29) is reported in the central panel of Fig. 5C looking at the solvent-exposed concave face of the structure. Finally, the spherical structure of 24-mer soluble HtrA is reported in the right-hand side of the figure. The regions recognized by anti-CM rHtrA and anti-CM rHtrA-OMV antibodies are highlighted in the 3 models maintaining the same colour code as in (B). The models were generated from PDB structure 3cs0.

To address whether CM rHtrA is found on the surface of the vesicles, we used flow cytometry and EM analyses. In the case of flow cytometry analysis, CM rHtrA-OMVs were incubated with anti-CM rHtrA antibodies followed by staining with fluorescence-labelled secondary antibodies. Upon antibody treatment, a consistent shift in the fluorescent intensity of OMVs was observed (Fig. 2C and 5S). OMV localization of CM rHtrA was also analyzed by immune electron microscopy using gold-labelled secondary antibodies. As shown in Fig. 2D, approximately 33% of OMVs (based on analysis of 7 EM fields containing 205 OMVs) were found associated with gold particles. This is consistent with the fact that since OMVs are constitutively released throughout bacterial growth, only OMVs generated after IPTG induction should be loaded with HtrA. Interestingly, similar to what Shen and colleagues (32) observed recently, the gold particles appeared to be confined in electron-dense protein material, which adhered to the surface of OMVs (Fig. 2D). A significant fraction of the gold particles were either not in direct contact with OMVs or were freely dispersed in the EM field (approximately 75% of all counted gold particles). While this might partly be due to a partial OMV rupture during sample preparation, the nature of HtrA interaction with OMVs deserves further investigation.

From these analyses, we concluded that delivering CM rHtrA to the *E. coli* periplasm allows its association with the OMVs and that the protein is at least partially exposed on the surface of OMVs.

### CM rHtrA-OMV induces anti-HtrA-specific antibodies in mice

We next analyzed whether CM rHtrA-OMV could induce HtrA-specific antibodies in mice. BALB/c mice (6 mice/group) were immunized with 3 doses (given at 2-week intervals) of 50 µg of CM rHtrA-OMV (total protein content) formulated in alum hydroxide, and anti-HtrA antibody titres were compared with titres elicited by 1 and 20 µg of CM rHtrA (3 doses in alum hydroxide).

As shown in Fig. 3A, CM rHtrA-OMV induced anti-HtrA-specific antibodies at titres that were only 3-fold lower than those obtained upon immunization with 1 µg of alum-formulated CM rHtrA, even though the amount of HtrA present in 50 µg of CM rHtrA-OMVs was estimated to be approximately 0.1 µg. This piece of data suggests that OMVs exert an adjuvant effect on rHtrA immunogenicity. We also tested whether OMV's adjuvanticity on rHtrA also occurred by adding 50 µg of “empty” OMVs to 1 µg of alum-formulated CM rHtrA. As shown in Fig. 3A, this was not the case in that empty OMVs addition appeared to decrease rather than increase in HtrA-specific antibody titres. The highest antibody titres were obtained by immunizing animals 3 times with 20 µg of alum-adjuvanted CM rHtrA (Fig. 3A). Finally, no anti-CM HtrA antibodies were detected when mice were immunized with “empty”, alum-formulated, OMVs derived from BL21(DE3) Δ*tolR(*pET), indicating that the endogenous *E. coli* HtrA (approximately 43% identity with CM-HtrA) did not induce antibodies cross-reacting with CM HtrA.

The specificity of anti-HtrA antibodies elicited by CM rHtrA-OMV immunization was further confirmed by Western blot analysis on total extracts from renografin-purified CM EBs (approximately 10^7^ EBs/lane). As shown in Fig. 3B, sera from mice immunized with 50 µg of CM rHtrA-OMV were able to detect HtrA protein in EB extracts, while sera raised against “empty” OMVs did not.

Finally, we analyzed the ability of anti-CM rHtrA-OMV antibodies to bind to the surface of chlamydial EBs by flow cytometry. As shown in Fig. 3C, the antibodies gave a shift in fluorescence intensity comparable to the one obtained using anti-CM rHtrA antibodies, whereas sera against “empty” OMVs were negative.

### Anti-CM rHtrA-OMV antibodies neutralize Chlamydia infectivity in vitro

With the experiments described above, we demonstrated that CM rHtrA-OMVs induced HtrA-specific antibodies. We next analyzed whether, in contrast to what was observed with anti-CM rHtrA antibodies, the antibodies induced by CM rHtrA-OMV could neutralize *Chlamydia* infectivity in vitro. Hence, we repeated the same in vitro neutralization assay described above by pre-incubating CM EBs with anti-CM rHtrA-OMV mouse sera. As shown in Fig. 4 (left panel), CM EB infection was significantly reduced and neutralisation titres were similar to those obtained with anti-CM rMOMP polyclonal antibodies used as a positive control. Anti-CM rHtrA-OMV antibodies were also able to cross-neutralize the infectivity of CT EBs, while anti-CM rMOMP antibodies, as expected for a protein which is species-specific, did not show any cross-neutralisation (Fig. 4, right panel).

One possible explanation of the functional difference between anti-CM rHtrA-OMV and anti-CM rHtrA antibodies is that the strong adjuvant effect of OMV might influence not only the quantity but also the quality of antibody response. However, immunization with rHtrA mixed with empty OMV in alum did not increase the neutralizing activity of the sera with respect to immunization with rHtrA alone (data not shown). A second interpretation is that anti-CM rHtrA-OMV and anti-CM rHtrA antibodies recognize different protein epitopes. To investigate this hypothesis, an epitope mapping analysis was carried out using anti-CM rHtrA-OMV and anti-CM rHtrA mouse sera. A total of 95 overlapping 15-mer peptides (10-residue overlap), spanning the amino acid sequence of the mature CM HtrA form, from amino acid 17 to 497, were synthesized on a cellulose membrane and peptide recognition profiles of the 2 sera were compared by dot blot analysis. As shown in Fig. 5, a clear difference was observed: while anti-CM rHtrA antibodies almost exclusively recognized 4 adjacent peptides spanning the HtrA sequence from amino acid 87 to 106 (included in Region 3 in Fig. 5B), anti-CM rHtrA-OMVs antibodies recognized a sequence that only partially overlaps with the anti-CM rHtrA target region, but interacted with 7 additional regions. Interestingly enough, the recognition pattern of anti-CM rHtrA-OMV antibodies was highly similar to the peptide recognition profile of a pool of sera from mice infected with CM EBs.

Overall, the epitope mapping analysis showed that the immunogenic profile of anti-CM rHtrA-OMV antibodies differed from that of the antibodies induced by CM rHtrA and mimics the epitope recognition of anti-HtrA antibodies induced by *Chlamydia* experimental infection in mice.

## Discussion

The good efficacy and safety profiles of the OMV-based licensed vaccines and our improved understanding of OMVs’ biology, biogenesis and unique adjuvanticity properties have stimulated the interest in OMVs as a platform for vaccine development. OMV-based vaccine development is moving along 2 major trajectories: vaccines based on OMVs from Gram-negative bacterial pathogens and used to prevent infections by the pathogens they derived from and vaccines based on recombinant OMVs decorated with heterologous antigens. The latter approach offers the possibility to design multivalent, easy-to-produce vaccines having built-in adjuvanticity. Another attractive aspect of the recombinant OMVs platform is that the expression of heterologous membrane-associated antigens, that is, proteins that require membrane interaction for proper folding, can theoretically be achieved preserving their native conformation. This would be an important property since functional antibodies targeting membrane protein antigens often recognize conformational epitopes.

In this work, we show that Chlamydial HtrA can be delivered to the OMV compartment and in doing so it folds in a conformation that elicits functional antibodies.

Our interest in Chlamydial HtrA was triggered by the fact that *Chlamydia* infection induces HtrA-specific CD4^+^ T cells both in humans and in mice (15). Furthermore, a combination of 4 Chlamydial antigens including HtrA elicited better protection in an experimental mouse model of *Chlamydia* infection (15). HtrA-mediated protection could theoretically involve the elicitation of both cell-mediated immunity and neutralizing antibodies since HtrA is exposed on the surface of Chlamydial EBs (24, and this work). However, as demonstrated here, anti-rHtrA antibodies did not prevent EBs from infecting epithelial cells in vitro. Since the recombinant HtrA used for immunization was expressed in the cytoplasm of *E. coli*, we thought that it could have a conformation partially different from the one assumed on the cell surface.

HtrA is an interesting protein from a structural standpoint. It is a multimeric periplasmic protein with a strong propensity to interact with phospholipids, such as phosphatidylglycerol (PG). Since phospholipids, PG in particular, constitute the bacterial inner membrane, it has been proposed that HtrA interacts with the periplasmic side of the inner membrane (19). Using a PG/DegP protein–liposome system, Skorko-Glonek and colleagues showed that membrane binding leads to slight changes in *E. coli* HtrA multimeric secondary structure with concomitant decrease in thermal stability and increase in proteolytic activity (18). More recently, Shen and colleagues elegantly demonstrated that, when on membranes, DegP appears as bowl-shaped oligomeric structures which assemble independently of the substrate. The authors propose that this new form of DegP, which features high proteolytic activity and low chaperone-like activity, may play vital physiological roles in vivo (32).

On the basis of all these pieces of information, we analyzed whether Chlamydial HtrA expressed in the *E. coli* periplasm could become associated with the OMVs and if so it could assume a conformation similar to the one present on the surface of chlamydial EBs, thus inducing neutralizing antibodies. Indeed, here we demonstrated that chlamydial HtrA associates with the OMVs, opening an interesting question on whether such association is a general feature in Gram-negative bacteria and, should this be the case, which might be its physiological role. Indeed, our proteomic analysis indicates that also the *E. coli* HtrA (DegP) is present in OMVs. More surprisingly, our immunogold electron microscopy analysis showed that the protein is at least partially localized on the surface of OMVs and seems to be organized in spherical particles that could resemble the bowl-shaped structure proposed by Shen and colleagues (29). Interestingly, while analyzing DegP localization in *Bordetella pertussis*, Baud and colleagues reported that the protein is also associated with the membrane, and their EM analysis of a high-molecular-weight fraction of purified *Bordetella* DegP appeared similar to what we observed in this work (33). Recently, Wu and colleagues showed that CT HtrA is detected as organism-free granules within the lumen of inclusions before being secreted into the cytosol of infected cells (22). Based on experimental evidences demonstrating the presence of vesicles inside and outside the chlamydial inclusion, the author hypothesizes that HtrA secretion occurs through a vesicle-mediated mechanism. Our results, showing the exposure of HtrA on the EB surface and indicating the tendency of HtrA from Gram-negative bacteria to associate with OMVs, would support the idea that HtrA is secreted by CT through a spontaneous OM blebbing and suggest an intriguing role of OMVs in the host-interaction mechanisms of this intracellular pathogen.

The mechanism through which DegP reaches the surface of OMVs remains to be elucidated. OMVs’ biogenesis envisages the budding out of the OM in areas where proteins linking the OM to peptidoglycan are absent. The accumulation of periplasmic proteins or the presence of curvature-inducing proteins should further augment vesiculation (2). However, the events leading to membrane fission still await additional studies. One possibility is that during the fission process DegP bound to phospholipids fuses to the OM. Alternatively, the phospholipid-bound DegP could be released out of the cell in proximity of membrane discontinuity occurring during the fission process and subsequently it interacts with the external side of the OMVs.

Whatever mechanism is involved, rHtrA-OMV has at least 2 interesting properties from a vaccine standpoint. First, it stimulates good levels of HtrA-specific antibodies, despite the fact that the amount of HtrA associated with OMVs is relatively low (approximately 0.1–0.2% of total proteins, corresponding approximately to 0.1 µg of protein per dose of vaccine used). This is in line with the notion that OMVs carry most of the PAMPs present in bacterial pathogens and so important for mounting robust adaptive immune responses. Interestingly, such adjuvanticity is not exerted if OMVs not carrying rHtrA are mixed with CM rHtrA before immunization. Indeed, HtrA-specific antibody titres were slightly reduced with respect to the titres elicited by immunizing animals with alum-formulated rHtrA in the absence of OMVs. The absence of an immunostimulatory effect of “empty” OMV on the elicitation of anti-CM rHtrA antibodies could be explained considering that for an adjuvant to exert its action, adjuvant/antigen codelivery to the same antigen presenting cell (APC) is usually required (34–36). Since the amount of total OMV proteins largely exceeds the amount of HtrA (50 µg vs. 1 µg) and since mixing recombinant HtrA to OMVs does not guarantee an efficient antigen (HtrA)/adjuvant (OMV) codelivery to the same APC (a situation that instead occurs with CM rHtrA-OMV immunisation), a large fraction of the antibody population elicited upon immunization with CM rHtrA +OMV is expected to be directed towards the OMVs’ proteins.

The second interesting property of CM rHtrA-OMV is that it induces functional antibodies, as judged by the ability to neutralize in vitro infectivity of EBs. This property has 2 implications. First, *Chlamydia* HtrA is somehow directly or indirectly involved in EB interaction with host cells, in line with our finding that the protein is surface exposed and second is that the conformation of OMV-associated HtrA resembles the one present on the surface of the EBs. Such conformation, which is likely to be mediated by HtrA interaction with lipid membranes, differs from the one assumed by the protein in the absence of lipids, as it is the recombinant protein purified from the cytoplasm of *E. coli*. The difference between the 2 conformations, which are both multimeric and enzymatically active, becomes indirectly evident by looking at the epitope recognition profiles of antibodies induced by immunization with CM rHtrA and CM rHtrA-OMV. While anti-CM rHtrA antibodies mostly recognize a region localized upstream and adjacent the proteolytic domain, anti-CM rHtrA-OMV antibodies also bind regions which map in the substrate-binding region (37) and the PDZ2 domain, the latter known to be involved in the formation of high-molecular-weight multimers (38). This is in line with the data reported by Shen and colleagues showing that the main differences between the oligomeric structures of membrane-free HtrA and membrane-associated HtrA occur at the intertrimer interfaces, where the PDZ1 and PDZ2 interactions appear to be weaker and more flexible in the bowl structure expected to be assumed upon membrane interaction, with respect to the spherical structure of the soluble multimeric HtrA (32). The different antibody profile elicited by CM rHtrA and CM rHtrA-OMV is further corroborated by our analysis of CM HtrA molecular model made on the crystal structures of *E. coli* HtrA. The modelling would indicate that the soluble spherical multimeric forms of CM rHtrA well expose the reactive region R3 (Fig. 5C), while the regions specifically recognized by anti-CM rHtrA-OMV antibodies (regions R5 and R6) are largely buried. In contrast, when we modelled the CM HtrA trimers in the lipid-associated, bowl-shaped structure proposed by Shen and colleagues (in Figure 5C the 4-fold symmetry bowl structure is shown), the R3 region tilts towards the bounded lipid membrane, suggesting why R3 region is less extensively recognized by anti-CM rHtrA-OMV antibodies, while R5 and R6 regions find themselves in the open chamber of the bowl structure, thus getting exposed to the solvent and becoming potentially accessible to antibody binding.

The mechanisms by which anti-CM rHtrA-OMV antibodies reduce *Chlamydia* infectivity in vitro remain to be elucidated. At least 2 possible explanations can be proposed. First, chlamydial HtrA is part of, or in proximity to, the component(s) recognized by the cellular receptor(s) involved in Chlamydial cell interaction and binding. In this case, the anti-CM rHtrA-OMV antibodies would decorate the HtrA associated with the EB surface much better than what the anti-CM rHtrA would do, thus preventing an efficient binding to the cell. Alternatively, should the proteolytic activity of HtrA be important for Chlamydial invasion/infectivity as proposed by Xu and colleagues (24), anti-CM rHtrA-OMV antibodies would efficiently inhibit the protease activity, thus impairing *Chlamydia* ability to infect cells. This latter hypothesis is currently under investigation.

The combination of rHtrA with other 3 *Chlamydia* antigens is protective and protection was shown to be largely mediated by IFNγ-producing CD4^+^ T cells (15). It will be interesting to analyze whether the replacement of rHtrA antigen with rHtrA-OMV further increases the protective activity of the antigen combination. Such increase could be mediated by the contribution of anti-HtrA neutralizing antibodies and by the strong adjuvant properties of OMVs.

For HtrA-OMV to become a component of a chlamydial vaccine, 3 aspects have to be addressed: (a) OMV production yield; (b) amount of HtrA in OMVs; and (c) OMV reactogenicity. Our data indicate that approximately 2 mg/L of OMVs are produced under laboratory conditions. We expect that such yields could be substantially improved in fermentation, as recently described by Berlanda Scorza and colleagues (39). Regarding HtrA relative concentration in OMVs, if required this could be increased either by modifying the promoter region and/or translation efficiency and RNA stability or by deleting some of the non-essential abundant proteins present in OMVs. We have recently estimated that 10 proteins account for approximately 50% of the total *E. coli* OMV protein content, and therefore the deletion of only few genes could be sufficient to substantially concentrate HtrA in OMV preparations (Grandi, personal communication).

Finally, it is known that OMVs can be reactogenic when injected in animals and humans and such reactogenicity is mostly due the presence of LPS, a component that plays an important role in adjuvanticity but whose concentration has to be carefully controlled. There are 3 main approaches to reduce LPS reactogenicity. First, OMVs can be treated with mild detergent to reduce LPS amount. Second, OMVs can be formulated with alum to absorb LPS. Third, it can be detoxified by genetically modifying its chemical structure. In this respect, a number of mutations have recently been described which substantially reduce LPS toxicity without impairing its adjuvanticity properties (40, 41).
